# A rare complication of lightning strike: Pulmonary contusion

**DOI:** 10.14744/nci.2020.24022

**Published:** 2020-12-31

**Authors:** Ayse Tolunay Oflu, Emre Kacar, Ayhan Pektas, Aysegul Bukulmez

**Affiliations:** 1. Department of Pediatrics, Afyonkarahisar Health Sciences University Faculty of Medicine, Afyonkarahisar, Turkey; 2. Department of Radiology, Afyonkarahisar Health Sciences University, Faculty of Medicine, Afyonkarahisar, Turkey

**Keywords:** Blastic injury, lightning strike, pulmonary contusion

## Abstract

Lightning strike is a devastating disaster, leading to various life-threating complications and even death. In lightning striking victims, high-voltage electric current can destroy many tissues and organs through various mechanisms. One of these mechanisms is the blunt trauma that injures the organs by creating a blast effect. Although not frequent, blunt trauma may result in various solid organ injuries such as pulmonary contusion. In this article, we reported a 15-year-old male patient who was admitted to the emergency department because of lightning strike in open terrain. Although he was conscious and vital signs were normal at presentation, respiratory distress developed on the 4^th^ day. Unilateral pulmonary contusion was detected on the computerized tomography of the thorax. The patient was treated with supportive oxygen and intravenous hydration therapy. His respiratory distress improved on the 6^th^ day and control posteroanterior chest radiograph revealed that pulmonary hemorrhage was spontaneously resorbed. On the 9^th^ day, he was discharged with normal respiratory findings. The patient did not have any complaints during the 3-month follow-up after discharge.

**L**ightning is a natural phenomenon containing energy between 100 and 300 million volt and generating heat approximately 3000°C [1]. The incidence of lightning strikes worldwide is estimated to be 0.09–0.12/100,000 people. Annual mortality due to lightning is reported to vary between 0.2 and 1.7 deaths per million people per year worldwide [2]. It is estimated that the number of deaths due to lightning strikes in Turkey is approximately 400 deaths/year [3].

Most of the death related to lightning strike are due to sudden ventricular fibrillation or asystole. Neurological complications, cardiac dysrhythmias, burns, tympanic, and ocular damage may develop in survivors frequently. Although rare, solid organ injuries may occur by the blastic effect of lightning [4]. The organs such as the spleen, liver, lungs, and bowels can be injured by blunt trauma due to shock waves or falling or being hit with an object [1].

In this article, we present a case of pulmonary contusion due to blastic injury of lightning strike.

## Case Reort

A 15-year-old male patient was admitted to the emergency department because of lightning strike. Other victims were the patient’s sister, his mother and father. During the incident, they were in an open air vehicle standing under heavy rain on the field. The vehicle was shaken after a severe flare, noise, and the patient found himself thrown about 3 m away.

**Figure 1.**
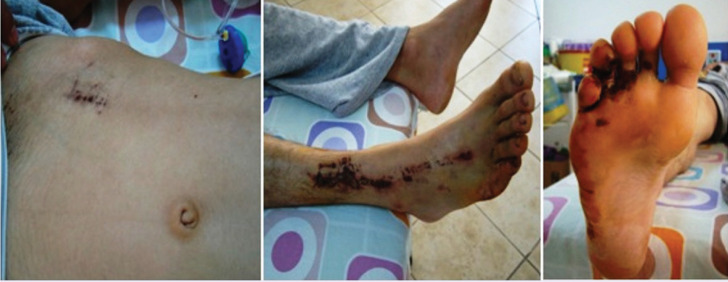
Photographs showing entrance wound, 1 cm × 3 cm in diameter, at 3 cm above the right groin, linear shaped second-degree burns extending along the lateral of the right ankle and dorsolateral of the right foot, exit wound, 1 cm × 1 cm in diameter, on the plantar region of the fourth toe of the right foot.

**Figure 2.**
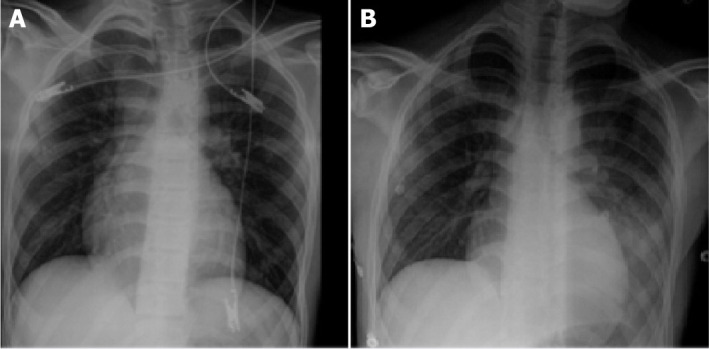
Patient’s initial PA chest radiograph **(A)** showing no pathological findings; patient’s 4^th^ day postero-anterior chest radiograph **(B)** showing heterogeneous density increment of lower zone of the left lung and blunting of the left costophrenic sinus.

On examination, he was conscious and remembered the event. His blood pressure was 118/58 mmHg, pulse rate was 105 beats/min and regular, and respiratory rate was 18 breaths/min. His physical examination revealed entrance wound, 1 cm × 3 cm in diameter, at 3 cm above the right groin. Linear-shaped second-degree burns extending along the lateral of the right ankle and dorsolateral of the right foot were detected. Exit wound was 1 cm × 1 cm in diameter and located in the plantar region of the fourth toe of the right foot (Fig. 1). Examination of other systems and laboratory tests on admission revealed no pathological findings. His arterial oxygen saturation (98%) and arterial blood gas analysis were also normal measured. No pathology was found in the initial electrocardiogram and posteroanterior (PA) chest X-ray (Fig. 2a).

**Figure 3.**
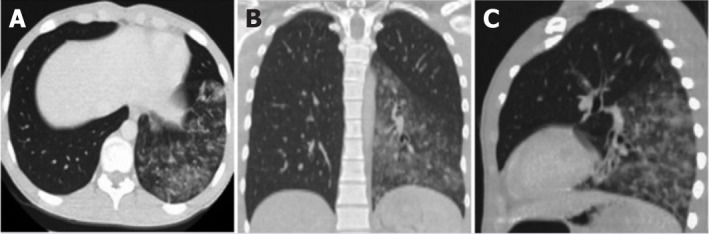
Axial, coronal and sagittal reformatted thoracic computed tomography images at parenchymal window showing ground-glass opacities and areas of consolidation, followed widely in the lower lobe of the left lung, compatible with pulmonary contusion and alveolar hemorrhage.

In the 4^th^ day of hospitalization, because of mild respiratory distress, PA chest radiograph was performed again. Heterogeneous density increment of lower zone of the left lung and blunting of the left costophrenic sinus were detected (Fig. 2b). Contrast-enhanced computed tomography (CT) of thorax showed ground-glass opacities and areas of consolidation, followed widely in the lower lobe of the left lung, compatible with pulmonary contusion and alveolar hemorrhage (Fig. 3). Oxygen therapy with face mask and intravenous hydration treatment was applied to the patient. No other medical treatment was needed. His respiratory distress improved on the 6^th^ day of hospitalization. Control PA chest radiograph revealed that pulmonary hemorrhage was spontaneously resorbed. The patient’s pulmonary symptoms improved rapidly and he was discharged on the 9^th^ day of hospitalization with normal heart, kidney, and liver functions. He did not have any complaints during the 3-month follow-up after discharge.

### Discussion

Lightning, violent, and sudden electrical discharge that occur between cloud and ground, descent down into the arms following a zig zag path [5]. Although exact mechanism of lightning injury is unknown, five types of mechanisms are foreseen. These mechanisms are as follows: First, a direct lightning strike; second, an indirect strike by contact with an object such as a tree; third, a side splash from a stricken object such as a metal bar; and fourth, mechanism is that a person could be injured by step voltages produced by lightning flowing through the resistance of the soil beneath. This ground current can also flow through up one limb and down another of the victim. Finally, the fifth mechanism is blunt trauma [6]. Based on laboratory experiments, barotrauma is also suggested as a sixth mechanism of lightning injury [7]. We think that our patient was affected by two of the anticipated mechanisms. First, lightning falls into a metal bar at the back of the vehicle, then electric current reached our patient by side splash and proceed in a linear pathway. Second, he was shot out from the vehicle, suggesting blunt trauma. We did not consider indirect strike due to the fact that our case did not contact with an object. The presence of the case on the vehicle during the lightning strike excludes the fourth mechanism, the ground current.

Pulmonary contusion is described as lung injury not accompanied by chest wall injury and caused by blunt chest trauma, explosion injuries, or a shock wave associated with penetrating trauma. In cases with pulmonary contusion, blood and other fluids accumulate in the lung tissue due to damage of alveolar capillaries but it does not involve a cut or a tear of the lung tissue. The severity and onset of clinical signs vary on a large scale and are not typical. Respiratory distress due to hypoxia and hypercarbia often develops slowly and peaks in approximately 72 h. Mild contusion may remain asymptomatic [8, 9]. Our case also had a mild pulmonary contusion and clinical findings were presented on the 4^th^ day. In cases of lightning strikes, symptoms may present late, as in our case and case of delayed esophageal rupture reported by Figgis et al. [4].

Blunt trauma caused by lightning strike may occur by two mechanisms. The first mechanism suggests that victims are shot out due to muscle contraction induced by electric current. Sudden and massive changes in the temperature create a blast wave in the second mechanism. Myocardial injury, pulmonary contusion, pulmonary rupture, major vessel rupture, intestinal rupture, tympanic membrane rupture, and ocular damage may develop due to blastic injury effect of lightning strike [5, 10]. For the 1^st^ time, Moulson [11] reported pulmonary contusion caused by blastic injury in a lightning strike case without any burn wound. Ohashi et al. [12, 13] suggested that the injuries such as intracranial hemorrhage, pulmonary hemorrhage, and solid organ rupture are related to neither fall nor current effect of lightning strike. In their clinical and experimental research, they showed that these injuries are due to the concussive effect of rapidly expanding steam produced by superheating water on the body surface by a surface flashover. They also extrapolated that the flashover causes voltage drop after the peak point and the time of voltage drop effects on mortality. If surface flashover is fast, it reduces the energy dissipation within the body and results in survival. We think that pulmonary contusion and hemorrhage were occurred by blastic effect of lightning originated from fast surface flashover in our case.

Direct strikes caused by lightning are potentially known as the most serious form of impact, as all energy passes directly through the victim’s body [14]. Direct strikes are likely to cause the most serious clinical complications [1]. Şener et al. [15] attributed the presence of bilateral pulmonary contusion and pleural effusion to direct strike due to the presence of multiple organ involvement in their lightning strike case. The presence of isolated pulmonary contusion and mild clinical findings in our patient suggests that the lightning strike did not occur by direct mechanism. In our opinion, our patient was protected against direct strike as the electrical current first has flown into the metal bar in the vehicle. We think that the metal bar reduced the energy voltage reaching to our case and minimized injury so that pulmonary contusion is limited in the lower lobe of the left lung.

### Conclusion

Lightning strikes threaten human life through different mechanisms such as blunt trauma. Victims catching the chance of survival may lose this because of various complications. In terms of late complications, it is very important to keep the patients under close follow-up as well as careful evaluation at admission.

## References

[1] Cooray V, Cooray C, Andrews CJ (2007). Lightning caused injuries in humans.. J Electrost.

[2] Pfortmueller CA, Yikun Y, Haberkern M, Wuest E, Zimmermann H, Exadaktylos AK (2012). Injuries, sequelae, and treatment of lightning-induced injuries: 10 years of experience at a swiss trauma center.. Emerg Med Int.

[3] Oztopal A (2019). Investigation of Turkey’s lightning observation.. Journal of Science and Engineering.

[4] Figgis P, Alvarez G (2012). Delayed esophageal perforation following lightning strike: a case report and review of the literature.. J Med Case Rep.

[5] Doğan KH, Demirci Ş, Günaydın G (2007). Deaths caused by lightning strike: Case report of three cases.. Genel Tıp Derg.

[6] Cooper MA (2002). A fifth mechanism of lightning injury.. Acad Emerg Med.

[7] Blumenthal R, Jandrell IR, West NJ (2012). Does a sixth mechanism exist to explain lightning injuries?: investigating a possible new injury mechanism to determine the cause of injuries related to close lightning flashes.. Am J Forensic Med Pathol.

[8] Ganie FA, Lone H, Lone GN, Wani ML, Singh S, Dar AM (2013). Lung contusion: a clinico-pathological entity with unpredictable clinical course.. Bull Emerg Trauma.

[9] Rendeki S, Molnár TF (2019). Pulmonary contusion.. J Thorac Dis.

[10] Mellor SG (1988). The pathogenesis of blast injury and its management.. Br J Hosp Med.

[11] Moulson AM (1984). Blast injury of the lungs due to lightning.. Br Med J (Clin Res Ed).

[12] Ohashi M, Hosoda Y, Fujishiro Y, Tuyuki A, Kikuchi K, Obara H (2001). Lightning injury as a blast injury of skull, brain, and visceral lesions: clinical and experimental evidences.. Keio J Med.

[13] Ohashi M, Kitagawa N, Ishikawa T (1986). Lightning injury caused by discharges accompanying flashovers--a clinical and experimental study of death and survival.. Burns Incl Therm Inj.

[14] Wankhede AG, Agrawal VR, Sariya DR (2010). An injury subjacent to lac ornament in a case of lightning.. Forensic Sci Int.

[15] Uzel Şener M, Demir A, Şener A (2019). Lightning-strike-induced acute lung injury: a case report.. Ulus Travma Acil Cerrahi Derg.

